# Host and HBV Interactions and Their Potential Impact on Clinical Outcomes

**DOI:** 10.3390/pathogens12091146

**Published:** 2023-09-08

**Authors:** Alexis Jose-Abrego, Sonia Roman, Saul Laguna-Meraz, Arturo Panduro

**Affiliations:** 1 Department of Genomic Medicine in Hepatology, Civil Hospital of Guadalajara, “Fray Antonio Alcalde”, Guadalajara 44280, Mexico; alexisjoseabiology@gmail.com (A.J.-A.); soniamariaroman@hotmail.com (S.R.); sslagna@gmail.com (S.L.-M.); 2 Health Sciences Center, University of Guadalajara, Guadalajara 44340, Mexico

**Keywords:** hepatitis B virus, HBV genotype H, immune response, metabolic interaction, clinical outcome, viral adaptation

## Abstract

Hepatitis B virus (HBV) is a challenge for global health services, affecting millions and leading thousands to end-stage liver disease each year. This comprehensive review explores the interactions between HBV and the host, examining their impact on clinical outcomes. HBV infection encompasses a spectrum of severity, ranging from acute hepatitis B to chronic hepatitis B, which can potentially progress to cirrhosis and hepatocellular carcinoma (HCC). Occult hepatitis B infection (OBI), characterized by low HBV DNA levels in hepatitis B surface antigen-negative individuals, can reactivate and cause acute hepatitis B. HBV genotyping has revealed unique geographical patterns and relationships with clinical outcomes. Moreover, single nucleotide polymorphisms (SNPs) within the human host genome have been linked to several clinical outcomes, including cirrhosis, HCC, OBI, hepatitis B reactivation, and spontaneous clearance. The immune response plays a key role in controlling HBV infection by eliminating infected cells and neutralizing HBV in the bloodstream. Furthermore, HBV can modulate host metabolic pathways involved in glucose and lipid metabolism and bile acid absorption, influencing disease progression. HBV clinical outcomes correlate with three levels of viral adaptation. In conclusion, the clinical outcomes of HBV infection could result from complex immune and metabolic interactions between the host and HBV. These outcomes can vary among populations and are influenced by HBV genotypes, host genetics, environmental factors, and lifestyle. Understanding the degrees of HBV adaptation is essential for developing region-specific control and prevention measures.

## 1. Introduction

Hepatitis B virus (HBV) infection remains a significant challenge for healthcare services worldwide. In 2019, over 296 million people had chronic hepatitis B, and 820,000 died due to hepatitis B complications. [1]. HBV is highly infectious and can be transmitted vertically from mother to child, and horizontally by sexual contact or contaminated materials [2]. While most HBV infections are asymptomatic, almost 90% of children infected and 5% of adults infected may develop chronic hepatitis B [2]. Without proper management, HBV infection can lead to serious liver complications, such as liver cirrhosis and hepatocellular carcinoma (HCC) [3].

In contrast to chronic hepatitis B, acute hepatitis B occurs within six months of exposure to the virus and refers to the initial phase of HBV infection [4]. Symptoms of acute hepatitis B include loss of appetite, fatigue, abdominal pain, jaundice, nausea, vomiting, fever, and dark urine [4]. While most individuals with acute HBV infection recover fully within a few months due to an efficient immune response that clears the virus [4], others may experience symptoms ranging from moderate to severe.

Fulminant hepatitis B is the most severe form of acute hepatitis B, affecting roughly 1–2% of patients with hepatitis B [5,6], and when it occurs, it can progress rapidly to life-threatening liver failure. Common complications include encephalopathy, coagulopathy, and multiorgan failure [7], attributed to the host’s exaggerated immune response that rapidly impairs the liver’s primary functions, such as detoxification, protein synthesis, and metabolism [7,8]. Dysfunction of these cellular processes can affect other organs (kidney, lung, heart, and brain), leading to abnormal personality changes, confusion, abnormal bleeding, easy bruising, prolonged bleeding time, and, in severe cases, coma [7,8]. Without specialized medical care, the survival rate of patients with fulminant hepatitis B is poor (~25%), and liver transplantation could be the best hope for survival [8].

On the other hand, occult hepatitis B infection (OBI) is characterized by the presence of low levels of HBV DNA in the liver or peripheral blood of people who test negative for hepatitis B surface antigen (HBsAg) [9]. In OBI, the virus can pass undetected within cells without causing significant liver damage [10]. This characteristic favors the long-term survival of both the host and HBV. However, under certain circumstances, such as immune suppression or chemotherapy, OBI can reactivate, leading to acute hepatitis B [11].

## 2. HBV Genotypes and Human Populations

HBV is classified into ten genotypes (A to J) and more than 40 sub-genotypes based on a genomic divergence of 8% and 4% (intra-genotype variation), respectively [12,13]. HBV sub-genotypes are typically designated using a combination of letters and numbers (for example, A1, A2, B1, B2, etc.). The classification of HBV in genotypes and sub-genotypes has important clinical implications, including their association with disease progression, response to antiviral therapy, and global distribution [12,13,14,15,16,17,18,19,20,21,22,23,24,25]. HBV genotypes A, B, C, and D were discovered simultaneously in 1988, and four years later, genotypes E and F were proposed [12,13]. Subsequently, genotypes G and H were discovered in 2000 and 2002, respectively [14,15]. In 2000, an aberrant genotype with evidence of recombination between genotypes A and C was identified [17]. These HBV sequences were classified as genotype I in 2008 [18]. Finally, genotype J was identified in an 88-year-old patient diagnosed with HCC in 2009 [19]. With the discovery of all HBV genotypes, it was possible to identify that each has a different geographical distribution and show a close relationship with its host populations [20,21]. Genotype A is prevalent in Europe and Eastern–Southern Africa. Sub-genotype A1 is found in southeastern Africa, Brazil, and South Asia. Meanwhile, A2, or Ae, is distributed in Europe and American countries with European ancestry. On the other hand, A3 is prevalent in central African countries near the Atlantic Ocean [26]. Genotypes B and C are commonly found in Asia, including China, Japan, Korea, and Australia. The greatest diversity of sub-genotypes B1–B9 is found in ethnic groups of the Indonesian archipelago. Interestingly, B6 in the North American Arctic suggests a complex evolutionary history [27]. Regarding the sub-genotypes C (C1–C5), most of them are distributed in Southeast Asia. However, C1 and C2 can also be detected in smaller proportions in Canada, the United States, and Northwestern Europe [26]. A study involving over 1000 genomes of HBV genotype D identified 11 sub-genotypes (D1–D11). The primary sub-genotypes are D1–D4. D1 predominates in the Mediterranean, Arab countries, India, Russia, Australia, and New Zealand. D2 prevails in Northern Europe, Eastern Europe, Southeast Asia, and North America, whereas D3 is detected in Europe, South Africa, the Caribbean Islands, and South America. D4 has been reported mainly in the Caribbean Islands with African ancestry [20,21,28]. Genotype E is predominant in West–Central Africa, while genotype F is widespread in Central and South America. [23]. The main F sub-genotypes are F1-F4, with F1 being predominant in countries near the Pacific Ocean, while F2 is detected in Brazil and Venezuela. F3 prevails in Colombia and Venezuela, and F4 is common in Argentina and Bolivia [23]. Genotype G is mainly detected in the risk group of men who have sex with men (MSM) from Mexico, the United States, France, and the Netherlands [23,24]. Genotype H is endemic mainly to Mexico [25]. Genotypes I and J have also been reported in Southeast Asia [18,19].

According to the region, HBV genotypes have been linked to several distinct clinical outcomes. In Spain, genotype A has a low antiviral response, while genotypes B and C have been associated with HCC in Asia [27,28]. In Native Alaskan populations, the sub-genotype F1 increased the risk of HCC 12-fold compared with genotypes B and D [29]. In Europe and Asia, genotype D has been linked with the worst rates of acute, fulminate, and chronic liver disease compared with genotype A [30]. In Mexico, patients infected with the genotype A2 were more susceptible to drug resistance mutations than non-A genotypes [31]. In a small population residing in the Brazilian Amazon, individuals with genotype D exhibited a 7.44-fold higher risk of developing advanced liver disease than individuals with genotypes A and F [32]. In contrast, most people are asymptomatic to the infection caused by genotype F1b in the Colombian Amazon region [33]. In Mexico, genotype H infection is often asymptomatic without significant clinical or laboratory manifestations of liver disease. Also, genotype H has been associated with low viral loads and OBI, particularly in the indigenous people of Mexico [10,23]. HCC is rare among Mexican patients with genotype H [34], but when found in another region, for example, in Japan [35], it is linked to HCC, suggesting that genotype H has sustained a closer adaptative relationship with the Mexican population. On the other hand, the clinical outcomes and diversity of HBV genotypes could be affected by the recombination and mixing of HBV genotypes [36,37]. Recombination occurs when two distinct HBV genotypes or sub-genotypes infect the same host cell, exchanging genetic material during replication [36]. Meanwhile, HBV mixture refers to the individual presence of more than one HBV genotype in the same host, which can occur due to co-infection or super-infection. Both recombination and HBV mixtures have been associated with several clinical outcomes, such as the development of liver fibrosis and hepatocellular carcinoma [37,38]. In patients with human immunodeficiency virus (HIV), mixtures of three HBV genotypes increased the risk of significant liver fibrosis 15-fold compared with dual-mixtures or single-genotype infection [39]. In addition, it was found that chronic patients with very high viral loads (>1 million IU/mL) are typical in the presence of HBV genotype mixtures [39]. High viral load levels may be due to the interaction between viral proteins and the genome of different HBV genotypes. A study showed that genotype G can use the core protein of genotype A2 to enhance its viral replication [40].

## 3. SNPs and HBV Clinical Outcomes

In addition to HBV genotypes, some variations in the patient’s genome, known as single nucleotide polymorphisms (SNPs), have been linked to different clinical outcomes, such as the risk of cirrhosis, HCC, OBI, hepatitis B reactivation, and clearance (Table 1). Cirrhosis-associated SNPs are in genes that participate in extracellular matrix production, immune response, intracellular adhesion, and signaling pathways [41,42,43,44]. A study in China associated SNPs at codons R241-E469 of the intercellular adhesion molecule-1 (ICAM-1) gene with decompensated cirrhosis [41]. Also, the haplotype A874 and A2109 in the gamma interferon (IFN-γ) gene increase the risk of cirrhosis in the Chinese population 1.5-fold [42]. A multivariate analysis found that Korean patients with detectable HBV viral load and the L/L genotype in the transforming growth factor (TGF)-b1 gene are at risk of developing cirrhosis [43]. In another Chinese study, the presence of the rs4796793C-rs2293152G-rs1053004T haplotype in signal transducer and activator of transcription 3 (STAT3) significantly increases the risk of cirrhosis [44].

Among the genes that increase susceptibility to HCC in chronic HBV patients are STAT4, complement component (C2), protein phosphatase 1 catalytic subunit beta (PPP1CB), tumor suppressor protein (p53), MDM2 proto-oncogene (MDM2), DEP domain containing 5 (DEPDC5), X-chromosome long arm band 22.1 (Xq22.1), and CD33 molecule (SIGLEC3 or CD33), which were found in studies conducted in Asian populations only [45,46,47,48,49,50,51]. Based on the odds ratio (OR) value, the most important SNPs associated with HCC in HBV Korean patients are rs1042522 in p53 and rs2279744 in MDM2, which increase the risk of HCC 3.59- and 4.27-fold, respectively [51]. Combined, the homozygotes MDM2C/C (p53: rs1042522) and G/G (MDM2: rs2279744) had a 20.78-fold higher risk of HCC than the rest of the genotypes [51]. 

Several SNPs have been analyzed in OBI cases and controls. However, only variations in HLA have shown a strong association with OBI. A study found that the T allele of HLA-DPA1 (rs3077) increases the risk of OBI 6.12-fold in the Indonesian population [52]. In northwest China, HLA-C*07:01, B*44:03, DRB1*07:01, and DQB1*02:02 were related to OBI with OR values of 4.7, 2.1, 2.0, and 1.9, respectively [53]. After achieving a sustained viral response, some HBV patients may develop reactivation, triggered by immunosuppressive therapy, autoimmune diseases, and organ transplants [54]. It has been reported that 51.9% of patients with chronic hepatitis B can present reactivation after antiviral treatment [55]. HBV reactivation can be silent, but in patients with cirrhosis, it indicates a poor prognosis [55]. Currently, the AA genotype of HLA-DPB2 (rs872956) is associated with HBV reactivation in Japanese patients treated with immunosuppressive therapy (OR: 8.27, *n* = 42) [56]. In lymphoma patients (*n* = 14), the SNPs in IL-13 (rs1295686) were associated with the reappearance of HBsAg in Chinese patients [57].

On the other hand, among the genes associated with spontaneous clearance of HBV infection are sodium taurocholate cotransporting polypeptide (NTCP), Toll-like receptor (TLR), interferon-induced helicase C domain-containing protein 1 (IFIH1), microRNA 219-1, IL, and HLA [58,59,60,61,62,63]. Of these, one of the most studied is the SNPs in NTCP; this protein is a transporter of bile acids in the liver and functions as an entry receptor for HBV. NTCP is encoded by the solute carrier family 10 member 1 (SLC10A1) gene [58]. A study in Taiwan found that the A allele SLC10A1 rs2296651 is associated with HBsAg seronegativity, while the AG or AA alleles are protective factors against HCC [63]. Based on a meta-analysis of nine case-control studies, the A allele of SLC10A1 rs2296651 shows strong evidence of spontaneous clearance [64].

**Table 1:** Single nucleotide polymorphisms (SNPs) associated with HBV clinical outcomes.

Clinical Outcome and Associated Genes	Population	SNP Database	OR	95% CI	Reference
Intercellular adhesion molecule-1 (ICAM-1)	China	rs1799969 (A)	4.197	2.550–7.074	[41]
Interferon gamma (IFN-γ)	China	rs2430561 (A) + rs1861494 (A)	1.485	1.065–2.070	[42]
Transforming growth factor (TGF)-beta1	Korea	rs1982073 (LL)	3.408	1.279–9.085	[43]
Signal transducer and activator of transcription 3 (STAT3)	China	rs4796793 (GG)	2.17	1.11–4.23	[44]
Signal transducer and activator of transcription 4 (STAT4)	China	rs7574865 (TG)	1.17	1.03–1.34	[45]
Complement component 2 (C2)	China	rs9267673 (TC)	1.37	1.15–1.63	[45]
Human leukocyte antigen (HLA)-DRB1	China	rs2647073 (CA)	1.63	1.29–2.06	[45]
Human leukocyte antigen (HLA)-DRB1	China	rs3997872 (AT)	1.86	1.32–2.62	[45]
Human leukocyte antigen (HLA)-DQ	China	rs9275319 (GA)	1.32	1.06–1.64	[45]
Hepatocellular carcinoma					
Protein Phosphatase 1 Catalytic Subunit Beta (PPP1CB)	China	rs13025377 (AA)	1.54	1.22–1.95	[47]
Mouse double minute 2 homolog (MDM2)	Korea	rs2279744 (G)	4.27	2.23–8.20	[51]
Tumor protein 53 (p53)	Korea	rs1042522 (Pro/Pro)	3.59	1.77–7.31	[51]
MDM2 + p53	Korea	rs2279744 (G/G) + rs1042522 (Pro/Pro)	20.78	5.25–82.36	[51]
DEP Domain Containing 5 (DEPDC5)	China	rs1012068 (CC)	2.397	1.251–4.595	[46]
X-chromosome long arm band 22.1 (Xq22.1)	China	rs5945919 (AG)	2.22	1.15–4.30	[49]
CD33 molecule (SIGLEC3 or CD33)	Taiwan	rs12459419 (C)	1.256	1.027–1.535	[50]
Occult hepatitis B					
Human leukocyte antigen (HLA)-DPA1	Indonesia	rs3077 (CC)	6.12	1.30–28.85	[52]
Human leukocyte antigen (HLA)-DPA1	Indonesia	rs3077 (T) + rs3135021(G) + rs9277535 (A)	4.9	1.12–21.52	[52]
Human leukocyte antigen (HLA)-DQB1	China	NA	2.15	1.118–4.161	[53]
Human leukocyte antigen (HLA)-HLA-C*07:01	China	NA	2.146	1.070–4.306	[53]
Human leukocyte antigen (HLA)-B*44:03	China	NA	4.693	1.822–12.086	[53]
Human leukocyte antigen (HLA)-DRB1*07:01	China	NA	1.919	1.188–3.101	[53]
Human leukocyte antigen (HLA)-DQB1*02:02	China	NA	2.012	1.303–3.107	[53]
HBV reactivation					
Human leukocyte antigen (HLA)-DPB2	Japan	rs872956 (AA)	8.277	1.540–51.550	[56]
Interleukin 13 (IL-13)	Taiwan	rs1295686 (AA)	4.683	1.030–19.156	[57]
Clearance or protection					
Sodium taurocholate cotransporting polypeptide (NTCP)	Korea	rs2296651(CT)	0.455	0.220–0.942	[58]
Toll-like receptor (TLR5)	Taiwan	rs5744174 (T)	1.32	1.03–1.69	[59]
Interferon-induced helicase C domain-containing protein 1 (IFIH1)	China	rs2111485 (G)	0.47	0.25–0.87	[60]
DExD/H-box helicase 58 (DDX58)	China	rs3824456 (C) or rs2074160 (A)	0.69	0.49–0.97	[60]
Sodium taurocholate cotransporting polypeptide (NTCP)	Taiwan	rs2296651(AA)	0.13	0.05–0.34	[63]

NA: Not available; letters in parentheses indicate allele or genotype; OR: odds ratio; 95% CI: 95% confidence interval.

## 4. HBV Life Cycle and Immune Response

**Figure 1:**
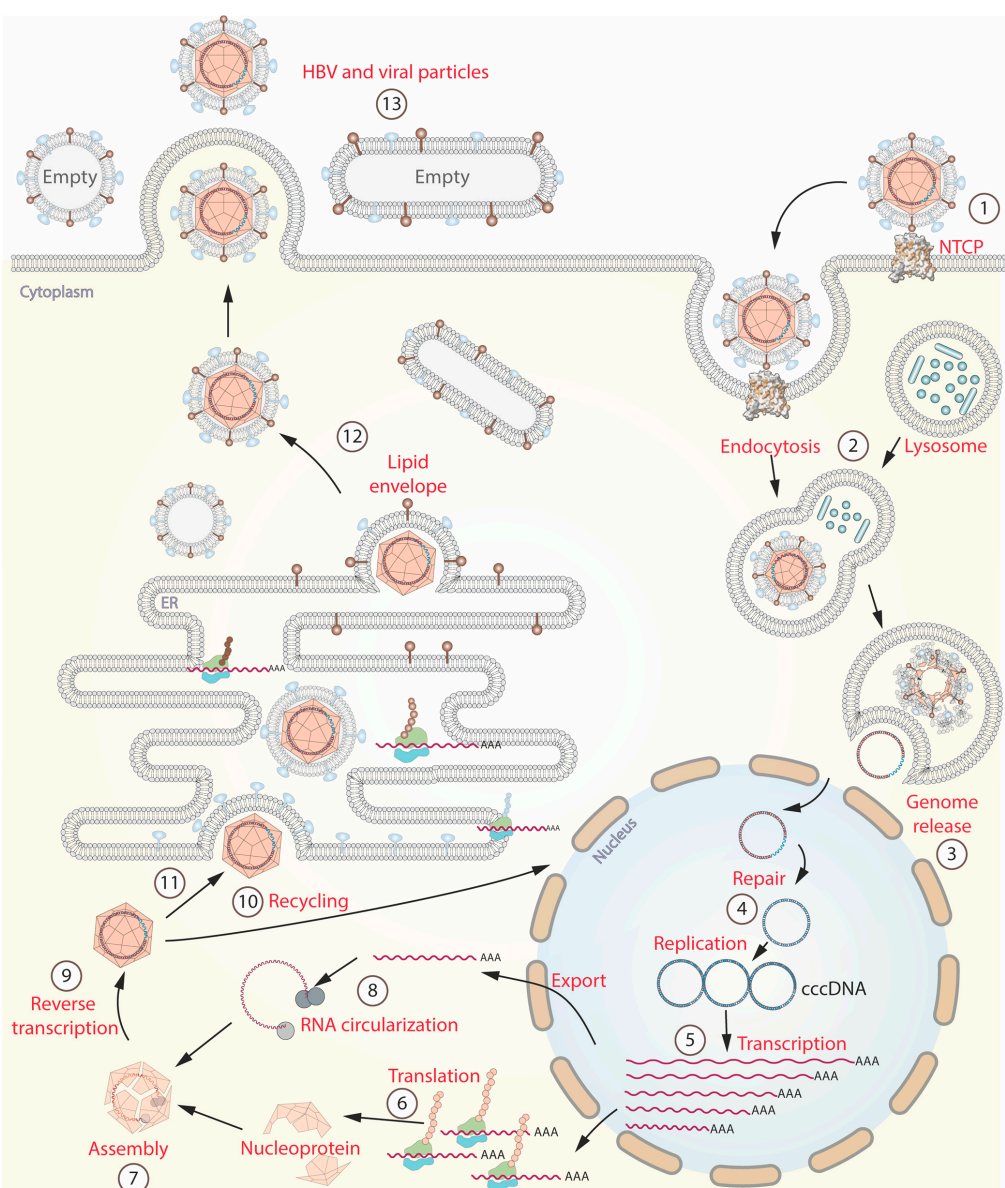
Hepatitis B virus life cycle. (1) The cycle begins with the specific binding of HBV to its receptor, sodium-taurocholate co-transporting polypeptide (NTCP). This binding triggers the process of receptor-mediated endocytosis. (2) Inside the hepatocyte, lysosomes break down the viral envelope and nucleocapsid. (3) Viral DNA is released into the cytoplasm. (4) Subsequently, the viral DNA is transported to the nucleus where it undergoes repair and conversion to a more stable form called covalently closed circular DNA (cccDNA). (5) cccDNA serves as a template for the synthesis of viral RNA. (6) Then, RNAs are transported to the cytoplasm and the rough endoplasmic reticulum (RER) to facilitate the production of viral proteins. (7,8) The core protein or nucleoprotein assembles around a circularized RNA within the cytoplasm. (9) After assembly, transcription is carried out by the viral polymerase. (10) Some viral particles are transported back into the nucleus to increase the pool of cccDNA. (11,12) Within the RER, the viral particles are enveloped by a lipid membrane, which carries hepatitis B surface antigen (HBsAg). (13) Finally, the mature virions are released into the bloodstream, accompanied by empty particles that serve as decoys for the immune response.

**Figure 2:**
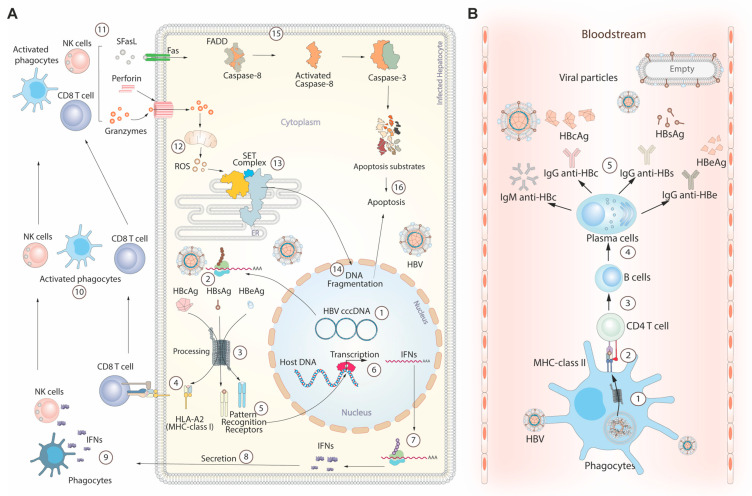
Immune response during HBV infection. (**A**) Cytokine-mediated hepatocyte death: (1) In this process, HBV cccDNA generates multiple RNA lineages. (2) These RNAs are transported to the cytoplasm to synthesize viral antigens, including core (HBsAg), surface (HBsAg), and e antigens (HBeAg). (3) Proteasomes cleave these antigens, creating peptides recognized by pattern recognition receptors. (5–7) Activation of these receptors triggers interferon transcription and translation pathways. (8–10) Secreted interferons activate resident phagocytes and natural killer cells (NK cells). (4) CD8 lymphocytes (CD8 T cells) can be activated by the major histocompatibility complex class I (MHC-I), specifically human leukocyte antigen-A2 (HLA-A2) subtype, presenting the peptides produced by the proteasome. (11) Immune cells produce cytokines that initiate apoptosis through cytoplasmic and nuclear pathways. (12) The nuclear pathway starts with perforin release, forming channels in the cell membrane for granzymes to enter the cytoplasm. Granzymes affect mitochondria, generating reactive oxygen species (ROS). (13) Elevated ROS levels trigger the release of the SET complex from the endoplasmic reticulum. (14) The SET complex contains endonuclease domains, facilitating DNA cleavage in the nucleus to aid apoptosis. (15) The cytoplasmic pathway is mainly initiated by the interaction between the soluble Fas ligand (SFasL) and Fas surface receptor (Fas) in the serum. This interaction involves recruiting adapter proteins like Fas-associated death domain (FADD). FADD then activates caspase-8, which activates downstream effector caspases, including caspase-3. (16) Caspases target specific proteins, called apoptotic substrates, which lead to cellular dismantling. (**B**) Viral particle neutralization in the bloodstream: (1) Viruses released from infected hepatocytes are captured and processed by bloodstream phagocytes. (2) Processed peptides are presented by MHC-class II to CD4 T cells, resulting in IL-21 release. (3–5) IL-21 induces B cell differentiation into plasma cells, producing primary antibodies against HBV infection. Abbreviations: IgM anti-HBc: immunoglobulin M antibodies against hepatitis B core antigen. IgG anti-HBc: immunoglobulin G antibodies against hepatitis B core antigen. IgG anti-HBs: immunoglobulin G antibodies against hepatitis B surface antigen. IgG anti-HBe: immunoglobulin G antibodies against hepatitis B e antigen.

The immunological reaction that follows HBV infection is a complex process that includes removing infected cells and neutralizing HBV in the bloodstream using antibodies (Figure 1). During the initial phase of infection, HBV enters hepatocytes (liver cells) through receptor-mediated endocytosis, utilizing the NTCP receptor [65]. Once inside the hepatocyte, the virus undergoes uncoating, releasing its DNA into the cytoplasm. The viral DNA is then transported to the nucleus and converted into covalently closed circular DNA (cccDNA), which can integrate into the host genome [66]. The HBV genome serves as a template for the transcription of several messenger RNAs (mRNAs), which are transported to the cytoplasm to be translated into proteins. Some of these proteins contribute to assembling new viral particles, while others are targeted for degradation within the proteasome [67,68]. The resulting peptides can be recognized by pattern recognition receptors (PRRs), initiating a signaling cascade that leads to the production of interferons (IFNs) (Figure 2A) [69]. IFNs activate immune response cells such as phagocytes and natural killer cells, while the peptides recognized by histocompatibility complexes class I (MHC-I) activate CD8 T cells [70,71]. These responses trigger the release of cytokines (granzymes, perforin, soluble Fas ligand, SFasL), which induce programmed cell death through cytoplasmic and nuclear pathways (Figure 2A) [72]. Simultaneously, circulating phagocytes capture and destroy HBVs in the bloodstream. In this case, the resulting peptides are presented by MHC-II to CD4 T cells [73]. This interaction stimulates the production of interleukin-21 (IL-21) in CD4 T cells, particularly in follicular helper T cells (TfH) [74]. IL-21 induces the expression of B lymphocyte-induced maturation protein-1 (BLIMP-1), which is a transcription factor key for plasma cell differentiation. Also, IL-21 promotes the generation of long-lived plasma cells in the bone marrow and continues to secrete antibodies for extended periods [75]. Among the most important antibodies produced by plasmatic cells are immunoglobulin (Ig) M and IgG, which neutralize the hepatitis B core antigen (HBcAg); IgG anti-hepatitis B e antigen (IgG anti-HBe), and IgG anti-hepatitis B surface antigen (IgG anti-HBs) (Figure 2B) [76]. In addition to neutralizing the virus, these antibodies are valuable for discerning between acute and chronic hepatitis B infections [77]. HBsAg, anti-HBc, and IgM anti-HBc is a distinctive pattern in patients with acute hepatitis B. However, after six months, the persistence of HBsAg and anti-HBc marks the transition toward chronic hepatitis B, indicating the body’s struggle to clear the virus [77]. This persistence might be attributed, in part, to HBV-mediated antagonistic effects that impair the host’s immune response and hinder the effective elimination of the virus [78]. One of HBV’s most important antagonistic effects is the inhibition of apoptosis through the repression of p53 by HBx [79]. Overall, the immune response plays a crucial role in the outcome of HBV infection, determining whether it is cleared or progresses to chronic infection. Furthermore, the balance between the immune response and the low levels of viral replication could promote the long-term coexistence of HBV and its host.

## 5. HBV and Metabolism

**Figure 3:**
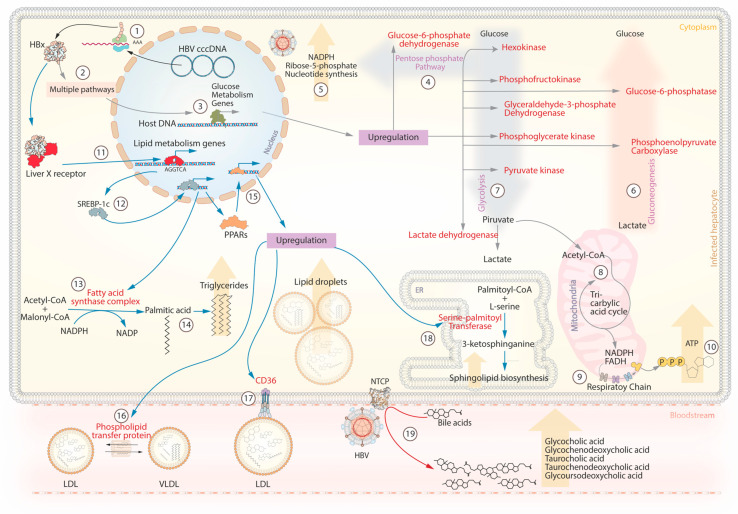
HBV interaction with host metabolic pathways. Metabolic control orchestrated by hepatitis B protein X (HBx): (1–3) The hepatitis B protein X (HBx) mediates metabolic control by activating genes linked to glycolysis (grey arrows). (7) HBx modifies the expression of long non-coding RNA UCA1, leading to increased expression of hexokinase and upregulation of phosphofructokinase, glyceraldehyde-3-phosphate dehydrogenase (GAPDH), pyruvate kinase (PK), phosphoglycerate kinase (PGK), and lactate dehydrogenase (LDH). This enzymatic surge propels enhanced glycolysis, elevating pyruvate production. (8) The resulting pyruvate enters the mitochondria, converting to acetyl coenzyme A (acetyl-CoA) and entering the Krebs cycle. (9) Citrate undergoes reactions releasing carbon dioxide and generating reduced compounds NADPH and FADH. (10) This process boosts succinyl-CoA conversion to succinate, producing more GDP that converts to ATP. (6) HBx influences gluconeogenesis by up-regulating phosphoenolpyruvate carboxykinase (PEPCK) and glucose-6-phosphatase (G6PC), facilitating the conversion of oxaloacetate to phosphoenolpyruvate and hydrolysis of glucose-6-phosphate to glucose. (4) HBx stimulates transcription of glucose-6-phosphate dehydrogenase (G6PD), pivotal in the pentose phosphate pathway (PPP). (5) The PPP crucially generates nicotinamide adenine dinucleotide phosphate (NADPH) and ribose-5-phosphate, vital for nucleotide biosynthesis. (11) HBx interacts with the liver X receptor (LXR) and increases the expression of sterol regulatory element-binding protein-1c (SREBP-1c). (12) SREBP-1c, a transcription factor, promotes the expression of genes essential for fatty acid synthesis, including fatty acid synthase (FAS) (15) and peroxisome proliferator-activated receptor (PPAR). (13) The cytoplasmic FAS complex transforms acetyl-CoA and malonyl-CoA into palmitic acid, using NADPH as a cofactor (14). Palmitic acid contributes to key fatty acids in triglycerides. (18) Overexpressed PPAR induces serine-palmitoyl transferase (SPT) expression, condensing serine and palmitic acid into ceramide within the endoplasmic reticulum, a critical component in sphingolipid synthesis. (16) PPARs initiate phospholipid transfer protein (PLTP) synthesis, enabling phospholipid transfer. (17) PPARs induce CD36 receptor expression, aiding fatty acid and lipoprotein uptake. (19) The interaction between HBV and NTCP disrupts bile acid metabolism, elevating serum bile acid levels.

The liver performs vital metabolic processes in the body, and hepatocytes are the primary target of HBV. Consequently, the HBV genome or viral proteins can inevitably interact at a molecular level with metabolic pathways. Evidence suggests that HBV can directly or indirectly modulate key enzymes involved in glucose metabolism, lipid metabolism, and bile acid uptake (Figure 3) [80,81,82,83,84,85,86].

An in vitro study showed that hepatitis B x protein (HBx) can up-regulate the expression of long non-coding RNA urothelial carcinoma associated 1 (LncRNA UCA1), which induces the expression of hexokinase [81,82]. In primary rat hepatocytes, phosphofructokinase transcription increased 72 h post-transfection with HBx [83]. A proteomic analysis found that the enzymes glyceraldehyde-3-phosphate dehydrogenase (GAPDH), pyruvate kinase (PK), phosphoglycerate kinase (PGK), and lactate dehydrogenase (LDH) were significantly up-regulated in HepG2 cells after transfection with different HBx genotypes [84]. These enzymes are crucial to carry out glycolysis in the liver. HBx can also affect gluconeogenesis through overexpression of phosphoenolpyruvate carboxylase (PEPCK) and glucose-6-phosphatase (G6PC), which catalyze the conversion of oxaloacetate to phosphoenolpyruvate and the hydrolysis of glucose-6-phosphate to glucose, respectively. Overexpression of these enzymes caused hyperglycemia in HBx transgenic mice [85]. In a yeast-based expression system, it was observed that the expression of preS2 mRNA (code HBsAg) may be reduced or suppressed when glucose concentrations reach 2% [86]. These findings suggest that the levels of HBsAg may be difficult to detect under high glucose conditions. HBx can also induce the transcription of the glucose-6-phosphate dehydrogenase gene (G6PD) [87,88], which is a key enzyme in the pentose phosphate pathway (PPP). The PPP is essential for generating nicotinamide adenine dinucleotide phosphate (NADPH) and ribose-5-phosphate, contributing to redox balance, energy production, and nucleotide biosynthesis [80].

In addition to its impact on glucose metabolism, HBV infection is associated with lipid droplet accumulation in hepatocytes. In cell culture, this phenomenon has been studied through the interaction of HBx with lipogenic genes, such as liver X receptor (LXR), sterol regulatory element-binding protein-1c (SREBP-1c), fatty acid synthase (FAS), and peroxisome proliferator-activated receptor (PPARs). In 2008, it was discovered that HBx can directly bind to the LXRα isoform [89]. Once activated, LXR is transported to the nucleus, where it activates the transcription of genes with the sequence AGGTCA [90]. Among them is the SREBP-1c, which acts as a transcription factor for genes responsible for synthesizing fatty acids, such as FAS and PPARs [91]. The FAS cytoplasmic complex converts acetyl-CoA and malonyl-CoA to palmitic acid, utilizing NADPH as a cofactor [91]. Palmitic acid is one of the most prominent fatty acids that contribute to the formation of triglycerides. On the other hand, PPAR upregulation can induce the expression of the enzyme serine-palmitoyl-transferase (SPT), which synthesizes sphingolipids [92]. Sphingolipids serve as structural components of the cell membrane and play a crucial role in maintaining the integrity of various organelles, including the endoplasmic reticulum, Golgi apparatus, mitochondria, and lysosomes [92]. Other enzymes induced by PPAR-α include carnitine palmitoyl transferase A1 (CPT1A), acyl-CoA dehydrogenase (ACADM), and peroxisomal acyl-CoA oxidase (ACOX1). These enzymes play crucial roles in β-oxidation, the process involved in the breakdown of fatty acids into acetyl-CoA molecules. Acetyl-CoA can enter the citric acid cycle or serve as substrates for the FAS complex [93]. Finally, the PPAR-ß/δ isoform can induce the expression of the CD36 receptor and the phospholipid transfer protein (PLTP). CD36 facilitates the uptake of fatty acids and lipoproteins, while PLTP is involved in the transfer of phospholipids between lipoprotein particles [93,94].

HBV can impact bile acid metabolism through its interaction with NTCP, a protein involved in the reabsorption of bile acids. NTCP serves as a receptor for HBV to enter hepatocytes [65,95], and this interaction can disrupt the normal uptake and recycling of bile acids within the liver. As a result, there can be an increase in the levels of bile acids in the serum of patients [96]. This symptom was analyzed in a cohort of patients with hepatitis B, finding that high levels of five bile acids (glycocholic acid, glycochenodeoxycholic acid, taurocholic acid, taurochenodeoxycholic acid, and glycoursodeoxycholic acid) were associated with the progression of cirrhosis [97]. Among the conjugated bile acids, there is a more significant elevation of taurocholic acid in patients with chronic hepatitis B compared with healthy controls [98]. These findings suggest that serum bile acid levels could be helpful as biochemical markers for fibrosis progression in patients with hepatitis B. HBV can modulate hepatocyte metabolism to create a favorable environment for its replication. HBx is the most important protein involved in the regulation of metabolism. The modulation of glucose metabolism creates an environment rich in glucose, energy, and nucleotides. At the same time, the control of lipid metabolism provides the necessary lipids to form new viral envelopes but also promotes the accumulation of lipid droplets in hepatocytes.

## 6. The Theory of Viral Adaptation

HBV-related viruses have been detected in various animal species, such as fish, amphibians, reptiles, rodents, bats, and primates [99]. The origin of these viruses can be traced back approximately 400 million years [99]. A multidisciplinary archaeological study recently identified traces of HBV genotypes in ten millennia-old human remains [100]. During this time, humans and HBV genotypes were subjected to selective pressures shaping their coevolutionary dynamics. Humans experienced different environmental conditions, infectious diseases, diets, and social interactions [101]. These pressures can influence human genetic diversity, metabolism, immunity, and infection susceptibility [101]. Simultaneously, HBV adapted to the selective pressures imposed by the host, leading to a complex interaction (HBV/human) that could determine its current distribution, epidemiology, and clinical outcomes.

**Figure 4:**
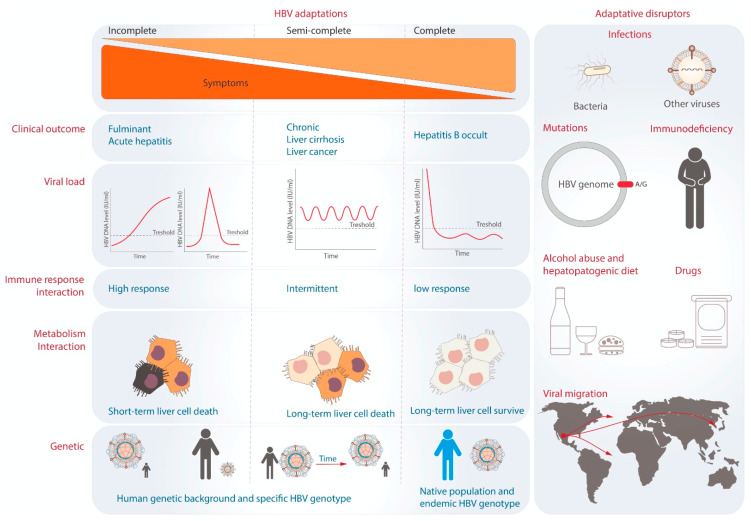
The theory of viral adaptation and adaptative disruptors. Three degrees of viral adaptation are shown on the left side. Each column indicates the representative clinical outcomes, viral load dynamics during infection, and immune and metabolic interactions. On the right, the potential factors that can break the levels of adaptation are represented.

In 2014, different degrees of adaptation were proposed for HBV and humans [102]. This hypothesis suggests that the severity of liver disease tends to be lower as the degree of adaptation increases (Figure 4) [102]. HBV–host adaptations can be grouped into incomplete, semi-complete, and complete [102]. The lowest degree of adaptation is known as “incomplete adaptation”. It is probably caused by a recent interaction between the virus and its host population [103]. Incomplete adaptation can manifest in two ways. Firstly, in cases of fulminant hepatitis, HBV infection triggers a hyperimmune response that severely impairs liver function. This state is considered ineffective for HBV survival because it relies on the host to exist. Fulminant hepatitis represents an extreme form of incomplete adaptation. Another form of incomplete adaptation is acute hepatitis B, where the immune response efficiently eliminates HBV DNA genomes without allowing the virus to develop a favorable intracellular environment for virus/host coexistence [6,7]. Because most acute infections are asymptomatic and self-limiting, people are often unaware that they are infected. The second degree of adaptation is “semi-complete adaptation”, which is observed in patients with chronic hepatitis B [102]. Although this infection does not immediately compromise the host’s life, prolonged exposure can lead to fibrosis, cirrhosis, and HCC [2,104]. Different HBV genotypes, particularly, genotypes B and C, confer a higher risk for HCC [28,29], indicating a potential disruption in the adaptive process regarding metabolic or immunological interactions. Finally, the highest degree of adaptation is exemplified by OBI [102], where patients have low HBV viral load (<200 IU/mL) and absence of HBsAg [9,10]. This state is likely achieved by integrating the HBV genome into host cells, stability of cccDNA, and immune tolerance [105]. It allows for a homeostasis state between the human host and the specific HBV genotype, resulting in asymptomatic infection for many years [102]. This type of adaptation has been observed in the native population of Mexico infected with genotype H [106]. Molecular dating estimates that genotype H has been present in the Mexican population for at least 2070 years [25]. Another virus with a similar adaptation may be the sub-genotype F1b in the Amerindian population of the Amazon basin [107]. These observations suggest that complete adaptation could develop between an endemic HBV genotype and its respective native population in each region [102].

The balance between viral replication and immune control is crucial for HBV to persist within the host without causing excessive liver damage. This balance can be disrupted by several factors, including viral mutations, a patient’s immune status, antiviral medications, exposure to toxins, co-infections, and lifestyle [108]. An additional significant factor to consider is the migration of HBV genotypes. As mentioned above, genotype H is less aggressive in native Mexican populations. However, when it migrates to a new population, such as Japan, it can cause acute hepatitis B [109] or liver cancer [35]. These data suggest the importance of identifying and avoiding the factors that act as adaptive disruptors in populations that have developed a high degree of adaptation between the host and the HBV genotype (Figure 3).

In contrast to HBV, the infection caused by hepatitis C virus (HCV) is considered more severe due to its high mortality rate (3.45 deaths per 100,000 people) compared with HBV (0.45 deaths per 100,000 people) [110]. Among the attributable fractions contributing to the incidence of liver cancer, 50% are related to hepatitis C, whereas 15% are related to hepatitis B [111]. Approximately 55–85% of adults with hepatitis C develop the chronic form, whereas less than 5% of adults with hepatitis B develop chronic infection [112]. This difference may indicate that spontaneous clearance is more common in acute HBV than in acute HCV infection. Based on the adaptation theory, it can be inferred from these findings that HCV exhibits a lower degree of adaptation than HBV, likely due to its recent introduction to human populations. A molecular dating study suggests HCV originated in the Old World approximately 3000 years ago [113]. However, HBV is estimated to have circulated in human populations for at least ten millennia [100]. The lower degree of adaptation of HCV to humans may explain why it is more likely to cause serious health problems, such as chronic infection, liver cancer, and death.

In conclusion, the clinical outcomes of HBV infection could result from complex immune and metabolic interactions between the host and HBV. These outcomes can vary among populations and are influenced by several factors, including HBV genotype, the genetic makeup of the population, exposure to toxins, co-infections, comorbidities, antiviral medications, diets, and lifestyle. The different types of HBV infection suggest three distinct degrees of viral adaptation. Understanding the degrees of adaptation in HBV genotypes among different populations is essential to develop the most appropriate control and prevention measures.

## Data Availability

Not applicable.
